# Mental Disorders as Risk Factors for New Onset Cardiovascular Diseases

**DOI:** 10.3390/biomedicines14051138

**Published:** 2026-05-18

**Authors:** Agata Anna Sakowicz-Hriscu, Oliwia Grunwald, Paweł Muszyński, Marcin Kożuch, Sławomir Dobrzycki

**Affiliations:** 1 Revit sp.z.o.o. Podlaskie Centrum Psychogeriatrii, Swobodna 38/8, 15-756 Bialystok, Poland; 2 Department of Invasive Cardiology, Medical University of Bialystok, Marii Sklodowskiej-Curie 24A, 15-276 Bialystok, Poland; 3 Department of Cardiology, Lipidology and Internal Diseases, Medical University of Bialystok, Żurawia 14, 15-569 Bialystok, Poland

**Keywords:** cardiovascular disease, mental disorder, psychiatric disorders, risk factors

## Abstract

**Introduction:** Cardiovascular diseases (CVDs) are a vast and widespread problem around the world, responsible for around one third of global deaths, of which 85% were due to heart attack and stroke in 2022. There are a lot of well-established risk factors for CVDs, including smoking, diabetes mellitus, obesity, poor diet, alcohol use, and sedentary lifestyle. Psychiatric disorders, however, are not among those frequently cited. Over a billion people worldwide suffer from some kind of mental disorder, with anxiety and depression being among the leading causes of long-term disability. All-cause death is significantly elevated in individuals with all mental health disorders. **Methods**: This narrative review aims to provide details on the selected psychiatric disorders and their pharmacotherapy with regard to the risk of developing cardiac illness by reviewing the available literature and the 2025 ESC Clinical Consensus Statement on mental health and cardiovascular disease. **Results**: Primary and secondary prevention of cardiovascular complications in the psychiatric disease population is an essential component in clinical healthcare. **Conclusions**: Taking all into account, it is essential to underline the role of the activation of the sympathetic nervous system and chronic inflammation, ultimately leading to metabolic syndrome in individuals with mental disorders, as well as an increase in residual cardiovascular risk and the development of CVDs, thereby worsening their long-term prognosis. In view of risky lifestyle behaviors in this population, it is essential to screen proactively, mitigate risk factors, consider the role of pharmacotherapy, and, if needed, initiate appropriate treatment.

## 1. Introduction

Cardiovascular diseases (CVD) have been described as the leading cause of mortality for over three decades [1]. The prognosis for the future CVD burden in the general population is expected to remain stable, with ischaemic heart disease as the main morbidity and high systolic blood pressure as the main risk factor [2]. Although there are multiple confirmed risk factors for cardiovascular diseases, varying by sex, age, and socioeconomic status, researchers rarely consider psychiatric health as one of them [3]. Psychological stress can be a contributing factor to the onset of cardiovascular disease through shared pathological pathways, including biological, inflammatory, genetic, and behavioral mechanisms. Disparities in the healthcare system, lower socioeconomic status, and risky behaviors (smoking, obesity, a sedentary lifestyle, and poor adherence to psychiatric treatment) complicate the screening and management of cardiac comorbidities [4,5].

Additionally, the hereditary and environmental factors in family history pose an increased risk of the development of psychiatric illnesses [6]. However, independent of familial burden, psychiatric disorders have been shown to promote CVDs, particularly in the first year after diagnosis [7]. In particular, severe mental illnesses, such as schizophrenia, bipolar disorder, or major depression, have been linked to worse cardiovascular outcomes, doubling the CVD mortality rate [8].

## 2. Literature Review

The literature search was performed on PubMed, Scopus, and the Web of Science database. It included publications between 2010 and 2026, with some individual papers being older, as they provided references. Medical Subject Headings (MeSH) such as ‘Mental Health’, ‘Mental disorders’, ‘Depression’, ‘Anxiety disorders’, ‘Feeding and Eating Disorders’, ‘Cardiovascular Diseases’, ‘Heart Disease Risk Factors’, ‘Myocardial Infarction’, ‘Cardiometabolic Risk Factors’, ‘Heart failure’, ‘Cardiovascular Risk’, ‘Biological mechanisms’, ‘Behavioral mechanisms’ and its’ combinations were used as a keywords. Articles, written in English and accessible online, relevant to the topic and methodologically adequate, were then included with the intention to cover the widest possible spectrum of the subject. Data extraction, including study population details, intervention, study design, and follow-up, was conducted by two independent authors. Applicable data derived from these articles was divided into subcategories, such as selected mental disorders, psychiatric pharmacotherapy, cardiovascular risk assessment, and prevention measures. This narrative review provides publications from varying standpoints to ensure a nuanced depiction of the topic at hand, mostly concentrating on randomized clinical trials, meta-analyses, and observational studies. The manuscripts that were non-English or not available in full text, as well as abstracts, posters, or unrelated to the research question, were excluded. Studies not addressing psychiatric factors or cardiovascular or mental health endpoints were also excluded. Ultimately, we selected 135 articles to be included in this review.

## 3. Depression

Depression is one of the most often diagnosed psychiatric illnesses worldwide, with an estimated 4% of the global population suffering from this affective disorder, more frequently appearing in women (6.9%) than men (4.6%) [9]. It is a stress-related affliction with stress being a common link between depression and somatic diseases, increasing the overall risk of cardiovascular, endocrine, and metabolic comorbidity [10].

There are multiple underlying mechanisms of the origins of major depressive disorder (MDD) that have a direct impact on the body’s homeostasis—desensitization of corticosteroid receptors due to prolonged stress, autonomic nervous system dysregulation, increased inflammatory response—all of which are commonly associated with CVDs, obesity, and diabetes [10] (Table 1). Elevated NT-proBNP levels are also an interesting finding in patients with MDD, setting the basis that depressive symptoms and CVD risk may be strongly interlinked [11,12,13]. Risk of thrombosis is also higher in depressed patients (with and without ischemic heart disease) due to elevated platelet activation [14].

Hypertension can be observed in the MDD group quite frequently, with the psychiatric disease doubling the risk of having a BP of 160/95 mmHg or higher in patients who have not been previously diagnosed as hypertensive [15]. Certain habits exhibited by depressed patients are contributing to poor health overall—these include lower vegetable and fruit consumption, low oral hygiene, sedentary lifestyle, and nicotine addiction [16,17].

Taking the above into consideration, it should not be surprising that depressive symptoms correlate with a higher incidence rate of cardiovascular diseases, with some sources citing this risk to be around 46% higher compared to the healthy control group [10,18]. Mortality rates are 1.44 times higher in the depressed CVD group, especially in men [19,20]. The presence of depression is also linked to the severity of CVD and is considered a contributing factor to worse patient outcomes and recurrent cardiovascular events [21]. Additionally, depression, more than anxiety, increases the risk of future CVD onset, which may be mitigated by adequate psychiatric treatment [22].

An analysis of 26 studies encompassing almost 2 million individuals shows that MDD heightens the overall risk of myocardial infarction (MI) by 1.28 times, stroke by 1.13 times, and heart failure (HF) by 1.04 times [19]. Depression at the time of acute MI is an independent risk factor of increased mortality post-MI, with lower heart rate variability (HRV) being looked into as a possible cause [23,24]. This illness often complicates the course of other non-psychiatric diseases that themselves elevate the risk of new-onset MI, increasing that risk even further—for example, diabetes type 2 coexisting with MDD presents an overall 82% higher incidence rate (IR) of MI compared to individuals without either, where that IR is only 30% higher in patients with only one of the two afflictions [25].

There is strong evidence that MDD is an important risk factor for stroke (ischemic, fatal, and total), with increased inflammatory response and platelet aggregation being possible interlinking mechanisms [26]. Whether depression can be considered a risk factor for hemorrhagic stroke, however, has to be studied further [26]. Both sexes have a similarly higher risk of stroke while suffering from depression [27].

Heart failure is more likely to occur in depressed patients, where the severity of their psychiatric symptoms proportionally correlates to the elevation of HF risk, with MDD also highly contributing to the development of other HF risk factors such as diastolic dysfunction and hypertrophy of the left ventricle [18]. Not only is depression considered an independent risk variable for HF development in susceptible groups, but it has also been linked to worse patient outcomes in those already suffering from HF [18,28]. Mortality rates are visibly higher in the depressed HF subgroup (21%) versus the non-depressed HF subgroup (15%) [29].

Coronary heart disease (CHD) is the most common type of heart disease, with approximately 5% of adults above 20 years of age living with the diagnosis [30]. With how prevalent both CHD and MDD are, they are bound to coexist in a certain group of patients, in which mortality and comorbidity rates are higher compared to non-depressed counterparts [31]. This number is even higher in women with CHD, since they are twice as likely to suffer from depression as men [32]. Not only is depression an independent risk factor for developing CHD, but the severity of depressive symptoms also correlates with a higher risk of new-onset CHD [33,34].

**Table 1:** Biomechanisms found in MDD as possible links to increased CHD risk [10,35,36,37,38,39,40,41].

Autonomic Nervous System Dysregulation	Biomarkers and Receptor Changes	Increased Inflammatory Response
increased sympathetic tone followed by enhanced catecholamine release decreased parasympathetic tone altered cardiac autonomic tone resulting in lower heart rate variability hypertension due to excessive and long-lasting release of stress hormones impaired vagal control, especially in those with suicidal ideation	desensitization of corticosteroid receptors due to prolonged activation of the limbic system possible hepatic insulin resistance due to chronic stress, leading to DM2 development increased platelet activation leading to possible thrombotic complications higher NT-proBNP levels correlate with the severity of depressive symptoms severity low BDNF expression impairs endothelial cells’ survival	promotion of proinflammatory cytokine release—IL-6, IL-1β, TNF, CRP decrease in production of anti-inflammatory cytokines—IL-4, IL-10 impaired anti-inflammatory function of corticosteroids a chronic rise in cortisol levels can lead to vessel constriction and subsequent vascular damage tissue inflammation due to excessive platelet degranulation

With many studies having been conducted and an interesting trend of multiple biomechanisms of both MDD and CVD overlapping, it is clear that depression must be treated as an independent risk factor for all new-onset CV events, but especially incident HF and sudden cardiac death [42]. It cannot be omitted in establishing a clinical prognosis for CVDs, as it worsens all cardiovascular outcomes. As to whether certain antidepressants alleviate that risk or, in turn, exacerbate it, it should be discussed and researched further.

## 4. Anxiety Disorders

Anxiety disorders are a group of mental diagnoses characterized by the extreme feeling of fear and panic, usually due to specific stressors (social anxiety disorder, agoraphobia, etc.) or no immediately identifiable threat (panic disorder). They tend to cause long-lasting stress and are chronic. All of these characteristics contribute to the prevalence of somatic comorbidity, including cardiovascular diseases [43]. Anxiety disorders have been named as an independent risk factor for cardiovascular health, increasing the risk of CHD by around 26% and its mortality rate, although, interestingly, their influence has not been as strong as that of depression, and no significant differences between sexes have been established [43,44,45]. Nevertheless, both new-onset and chronic anxiousness were linked to more frequent CVD diagnoses, with the latter being a risk factor only in men. In contrast, chronically anxious women did not seem to be majorly predisposed to CVD development [46]. There is other evidence that acute anxiety, compared to chronic, plays a bigger role in females, with phobias putting them at a higher risk of fatal CHD, specifically SCD [47].

The risk of MI and HF is also elevated in patients with anxiety disorders, regardless of their type [43,48]. Generalized anxiety disorder (GAD) is a predictor for adverse future cardiovascular outcomes, alongside other more well-known factors, such as left ventricular ejection fracture below 40% and older age [43]. It is also essential to note that this study found this to be true, accounting for post-MI depression, underlining the role of GAD in CVD risk calculation [43]. Different research supports this claim, where anxiety has been established as a risk factor for incident MI independent of coexisting depression [49]. Apart from MI, pathological anxiousness raises the risk of all CVD events according to meta-analyses—CHD by 41%, stroke by 71%, and HF by 35% [50]. This can be partially dictated by a high prevalence of hypertension in anxious patients, with around 38% of them having abnormally high blood pressure [51,52]. Other explanations may lie in the fact that anxiety, just as depression, is a stress-related disorder and shares numerous biological pathways with MDD [53]. Reduced HRV and vagal tone in anxious patients is associated with worse cardiological outcomes [54,55]. Changes in autonomic cardiac regulation may be one of the most important underlying mechanisms in this case [56]. Due to chronic activation of the sympathetic nervous system, a down-regulation in adrenergic receptors occurs [54].

## 5. Eating Disorders—Anorexia Nervosa and Bulimia Nervosa

Eating disorders (ED), along with substance abuse disorders, rank at the top when it comes to all-cause mortality rates in psychiatric illnesses and substantially increase the risk of suicide in women [57]. Cardiovascular complications are often associated with ED, which mostly stem from serious electrolyte imbalance and hemodynamic stress, manifesting as brady- or tachycardia, LV mass reduction, valve abnormalities, and cardiomyopathy [58]. Patients with anorexia nervosa (AN) were found to have an overall higher risk of major adverse cardiovascular events (MACE) and any CVD compared to the control group, with conductive disorder being the most common, at the same time having no major predisposition towards selected complications like stroke or atherosclerosis [59,60] (Table 2).

Mitral valve prolapse is one of them and can often be reversed with weight gain, although it is still not entirely clear whether it is mainly caused by LV mass reduction or low HRV, with some studies citing the latter as a more significant factor [49,61]. The most serious manifestation of a CV complication arising from EDs is cardiac arrest, although AN patients are just slightly more likely to experience it, and it was not linked to the changes in QTc interval, which are frequently described in AN [59,62].

## 6. Severe Mental Illness—Schizophrenia, Bipolar Disorder and Other Psychoses

Life expectancy in patients with psychotic disorders is markedly reduced by up to 20 years. Considering this, individuals diagnosed with psychotic disorders, classified as severe mental illness (SMIs), are more likely to develop cardiovascular diseases over the long term (e.g., during 10- and 30-year follow-up periods), particularly when at least two major cardiovascular risk factors are present. Studies have shown that the highest CVD risk occurs in patients with schizoaffective disorder, followed by those with bipolar disorder and schizophrenia. In comparison to the healthy population, the burden was the highest in smoking, hypertensive, diabetic, or obese female psychiatric patients aged between 18 and 59. Later on, the difference diminished with age. Additionally, compared to healthy individuals without SMI, younger patients with SMI show a higher prevalence of diabetes and obesity, as well as increased use of statins. Overall, well-established cardiovascular risk factors significantly contribute to the deterioration of health status in individuals with SMI and remain untreated across psychiatric diagnoses. Unfortunately, the probability of receiving proper regular screening for cardiac diseases is low [63,64,65,66,67,68,69].

However, in the 3-year period after the first episode of psychosis, the risk of death from heart disease did not differ from that of the general population [70].

Moreover, the genetic background of SMI, the presence of cardiovascular risk factors, and metabolic disturbances interlink, promoting chronic inflammation and the development of CVD comorbidities [71]. Abnormalities in glucose metabolism, adipokine profiles, visceral adiposity, and increased waist-to-hip ratio may lead to cardiometabolic complications. Genetic factors, persistent activation of the inflammatory pathways, abnormal pain threshold, and decreased pain sensitivity can be responsible for behavioral and metabolic changes in those individuals [72,73].

When considering coronary artery disease (CAD) and congestive heart failure, the risk is significantly elevated, particularly in association with antipsychotic use, higher body mass index (BMI), and pre-existing cardiovascular disease [65,74]. Interestingly, the coronary artery calcium (CAC) score, used for the preliminary diagnosis and severity assessment of CAD, does not differentiate between SMI and non-SMI patients despite the increased cardiovascular risk [75]. Although due to a possibly asymptomatic course of the disease, the first presentation might have been an acute myocardial infarction (AMI). Notably, AMI occurs approximately ten years earlier in patients with schizophrenia and is associated with worse prognosis and outcomes [76,77]. In both acute and chronic coronary syndromes, studies indicate that patients with SMI are less likely to receive revascularization procedures and secondary preventive measures, which contributes to higher mortality rates [78,79]. The probability of omitting CVD in the SMI population through primary care specialists or other specialists is very likely, especially in the schizophrenic group or in women with bipolar disorder.

Up to 66% of patients with schizophrenia were more likely to be underdiagnosed before cardiovascular death, despite the elevated occurrence of stroke and heart failure, especially in women aged from 41 to 50 [80,81,82]. Disparities in medical care access or quality of care were said to be one of the reasons behind elevated mortality rates in SMI patients [83]. A randomized clinical trial demonstrated that a comprehensive healthcare program, including outpatient behavioral counseling, significantly reduced the estimated 10-year risk of cardiovascular events. This reduction was attributed to lifestyle modifications, such as smoking cessation and appropriate management of hypertension and dyslipidemia [42]. The PRIMROSE BMI and lipid CVD risk prediction models of the first cardiovascular event were more accurate in SMI compared with models that include only established CVD risk factors [84].

This may explain why the probability of chronic coronary syndrome increases with a rising number of hospitalizations and advancing patient age. However, the sample size of this research was limited to 41 cases and is not necessarily representative of the population [85].

Taking all the above into account, according to the 2025 ESC Clinical Consensus Statement on mental health and cardiovascular disease, there is a special need to recalibrate CVD risk scores for patients with SMI [42].

## 7. Other Biological Mechanisms Connecting Psychiatric Diseases to Cardiovascular Risk

Mental stress-induced endothelial dysfunction led to a significant increase in adverse cardiovascular outcomes in patients with coronary artery disease. An imbalance between free radicals and antioxidant mechanisms, causing oxidative stress, significantly increases cardiovascular and psychiatric risk. Excessive production of reactive oxygen species may lead to the destruction of DNA, lipids, and proteins, causing subsequent alterations in cellular function, endothelial dysfunction, and inflammation, thereby influencing cell growth, proliferation, migration, and death. Oxidative stress and endothelial damage are well-established mechanisms in CVD development, resulting in vasoconstriction, procoagulant and proinflammatory states, particularly contributing to atherosclerosis, hypertension, and heart failure.

The brain has high oxygen consumption and low antioxidant reserves; thus, it is very sensitive to oxidative damage, resulting in subsequent neurotoxicity associated with neurodegenerative disorders, anxiety, and other psychiatric conditions [86,87,88,89,90,91,92,93]. Moreover, the neuroimmune axis is significantly influenced by brain-derived neurotrophic factor (BDNF), which acts as an essential growth factor for neuronal plasticity. It has been demonstrated that abnormal levels of BDNF may contribute to the neurodegenerative process and psychiatric disorders [94,95].

**Figure 1:**
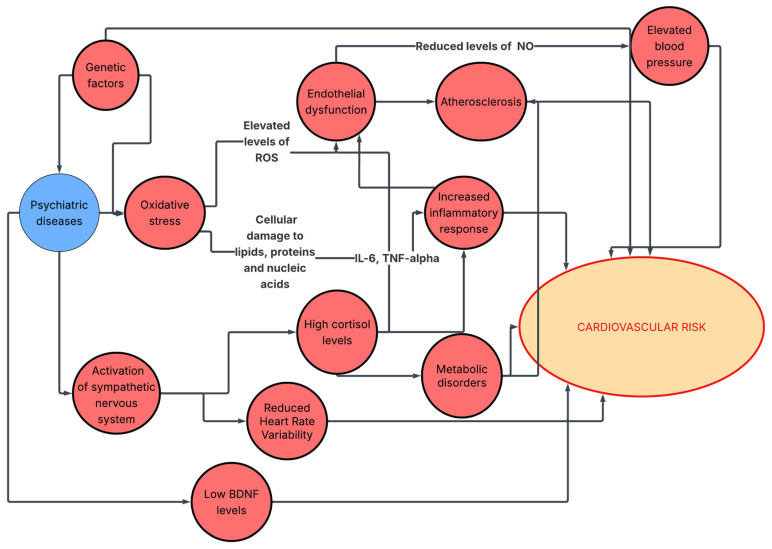
Summary of biomechanisms connecting psychiatric disease to cardiovascular risk. ROS—reactive oxygen species, IL-6—interleukin-6, TNF-alpha—tumor necrosis factor alpha, NO—nitric oxide.

Growing evidence suggests that disruption of the BDNF signaling pathway is also closely associated with the development of cardiovascular diseases (CVD), particularly through cardiac remodeling [96,97]. This cardio-neuronal damage has been linked to coronary artery disease, heart failure, cardiomyopathy, hypertension, and metabolic syndrome [98]. The summary is presented in Figure 1.

## 8. Pharmacotherapy in Psychiatric Disorders and Their Effects on CVD Risk

Medications used in psychiatry have multidirectional impacts, including a vast range of cardiovascular side effects. Therefore, selecting the optimal course of therapy for psychiatric disorders in at-risk patients is a crucial step that can either alleviate CVD symptoms and mitigate cardiological outcomes, or exacerbate them.

Even though depression is a recognized CVD risk factor, its adequate treatment can have a positive impact on the heart. Interestingly, both SSRIs and TCAs lowered the risk of MI, with the risk being slightly lower for women than men [99]. When it comes to antidepressant use in heart failure, there are conflicting results. Among the SSRI group, sertraline was found not to have any significant effect on death from cardiovascular causes or functional NYHA class, while escitalopram reduced cardiovascular mortality [100]. Fluoxetine demonstrated neutral or beneficial cardiac effects in some studies, though increased mortality was reported elsewhere [101,102]. In the SNRI group, venlafaxine might even lower the risk of HF, and duloxetine has not been linked to negative cardiac outcomes [103,104,105]. TCAs combined with beta-blockers have been associated with lower mortality rates among HF sufferers compared to the SSRI group, which was linked to poorer outcomes and non-adherence in some studies [106].

SSRI and TCA use was not linked to any adverse coronary events in depressed patients, with SSRIs even having the potential to reduce CHD risk. However, other studies show that TCAs do, in fact, increase mortality rates among those already treated for CHD and increase the 10-year risk of the first CHD event [35,107].

Overall, SSRIs, as the newer-generation drugs, are considered much safer than TCAs and may have potentially cardioprotective properties; hence, they are recommended as a first line of treatment (excluding high doses of escitalopram and citalopram). Unfortunately, the use of SSRIs and TCAs was also positively correlated with an increased risk of stroke, which was 24% and 34% higher, respectively [108,109]. TCAs like nortriptyline or amitriptyline can cause a QTc prolongation in healthy individuals, so their use ought to be limited and approached with caution. Although there is data confirming possible positive implications of TCA use in CVD patients, depending on their cardiological treatment, the risk of adverse effects should favor SSRI or SNRI usage [110]. Altogether, SNRIs have a more neutral effect on the heart; therefore, they can be used as a second-line therapy or when SSRIs are contraindicated, as mentioned in Table 3.

Even though benzodiazepines (BDZ) are one of the most effective anxiolytics, they have been associated with an increased risk of CVD and mortality. However, short-term, low-dose use may provide benefit in conditions such as silent ischemia or hypertension [118,119,120,121]. This most likely suggests that these factors are proportional to the CVD risk: the longer the period of BDZ/Z-hypnotics prescription and the higher the dose, the lower the cardioprotective influence and the higher the risk. The ESC 2025 consensus advises against BDZ first-line use as fast-acting anxiolytics, especially in older adults [42]. All in all, antidepressants are generally preferred as first-line therapy for anxiety disorders and PTSD.

Antipsychotic drugs have a variable cardiotoxic profile depending on dosage, duration of treatment, and concomitant use with other medications. CVD risk is usually the highest at the start of treatment, plateauing with higher doses [122]. However, according to the 2025 ESC clinical guidelines, the use of depot-form and second-generation antipsychotics, including monotherapy, lowers all-cause mortality, with the best results being achieved in patients receiving long-acting injection (LAI) olanzapine, oral clozapine, and oral flupentixol. Nevertheless, olanzapine and clozapine may increase the risk of metabolic syndrome, so their use should be carefully considered [123]. LAIs, especially paliperidone, presented with better metabolic outcomes than clozapine, while ziprasidone, cariprazine, and lurasidone have relatively safer metabolic profiles [122]. QTc interval prolongation is also something to be mindful of, since some newer antipsychotics, mainly ziprasidone and sertindol, may increase the risk of sudden cardiac death [124]. In at-risk patients, lurasidone might prove to be a better choice, showing the lowest risk of QTc prolongation [125].

First-generation antipsychotics are generally regarded as less cardiologically safe, with haloperidol considered as being the most problematic due to its association with acute ischemic heart disease, especially in women [122,126]. They also cause QTc prolongation more often than their newer counterparts [124]. This can cause some confusion, but it is still generally recommended to opt for the atypical ones, being aware of their aforementioned clinical implications. Additionally, before initiating antipsychotics with known and proven cardiotoxicity in at-risk patients, it is advisable to order an ECG and ask about the family history of SCD.

Taking the above into account, current evidence suggests that psychiatric pharmacotherapy has a positive impact on CVD risk mitigation. Although many nuances do exist and clinicians need to be aware of numerous limitations in certain clinical settings, as outlined in Table 4, it has to be underlined that untreated psychiatric disorders most probably carry a much greater CVD risk, and a multidisciplinary approach is recommended when managing high-risk patients.

## 9. Mental Disorders’ Role in Current Cardiovascular Risk Assessment

According to current guidelines, the SCORE2 and SCORE2-OP algorithms remain the main tools for CV risk assessment in patients without atherosclerotic cardiovascular diseases or severe metabolic diseases. The algorithm’s evaluation relies on blood pressure, lipid profile (non-HDL-C), sex, and smoking status. However, the guidelines suggest that certain factors influence the risk, increasing it beyond the estimations. These include the increased lipoprotein (a), systemic inflammation, stress symptoms, psychosocial stressors, social deprivation, and major psychiatric disorders. These factors should be used in the decision-making process regarding non-pharmacological and pharmacological interventions [130]. However, a holistic assessment, including unclassical factors, is still rarely used; the main factors influencing the clinical decisions are classical risk factors [131]. Awareness of the impact of mental disorders, or psychotropic drugs, on cardiovascular risk and metabolic function should be increased. Additionally, mental disorders should be included in risk stratification algorithms.

## 10. Prevention Measurements

The ESC Clinical Consensus Statement on mental health and cardiovascular disease highlights the bilateral relationship between mental disorders and cardiovascular diseases. Cardiovascular events can influence mental health, leading to an increase in anxiety, depressive symptoms, or post-traumatic stress symptoms in people with CVD, influencing the cardiovascular outcome and adherence to treatment. The consensus clearly states that clinical practitioners should perform screening for mental health problems (e.g., Generalized Anxiety Disorder GAD-7; Patient Health Questionnaire PHQ-9 or other validated tools), offer support, and, in cooperation with the patients and their families/caregivers, address the patients’ needs. In some cases, when the screening result suggests a moderate-severe mental problem, the patient should undergo psychiatric consultation [42].

**Figure 2:**
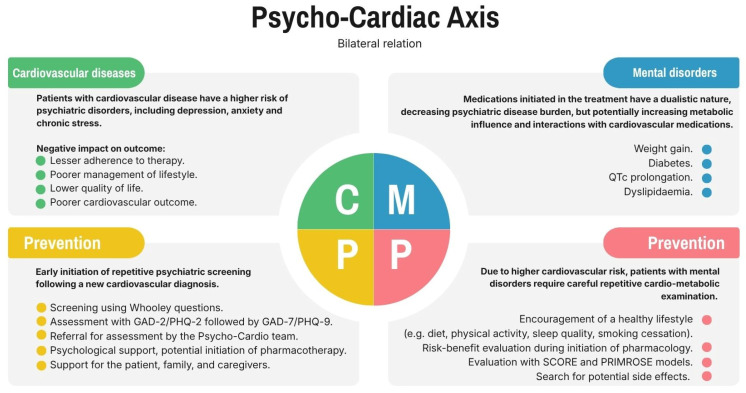
Summary of the potential tools used for prevention.

Mental disorders themselves increase cardiovascular risk, and additionally, many psychotropic medications may interact with cardiovascular drugs or negatively affect metabolic function. The most frequent of these negative effects include weight gain, hyperglycemia, hypertension, and lipid disorders. Those factors should be monitored during treatment. General preventive measures, such as lifestyle interventions such as physical activity, diet, smoking cessation, stress management techniques, and sleep hygiene, should be implemented in all patients with mental disorders or cardiovascular diseases. Moreover, when side effects persist, antipsychotic dose reduction or a change to a medication with a more neutral metabolic profile should be considered [42]. The data regarding treatment of dyslipidemia with severe mental illnesses is limited; the study by Blackburn suggests that statins improve lipid profile but do not affect cardiovascular risk [132]. Additionally, small studies suggest that interactions with cardiovascular medication, including statins, can exacerbate side effects (e.g., statin myalgia). However, those matters should be the subject of large randomized clinical trials [133]. The summary of the potential tools used for prevention is shown in Figure 2.

## 11. Conclusions

With such high prevalence rates of both CVDs and psychiatric disorders, it is crucial to note that they often coexist and impact each other’s clinical outcomes. Mental illnesses have numerous multidisciplinary complications, often influencing the heart and vascular system. Psychiatric medications are also well known to have numerous cardiologic implications. Multiple biological and chemical pathways appear to interlink the two conditions, contributing to chronic inflammation and activation of the sympathetic nervous system. In particular, metabolic syndrome—resulting from both the underlying disease and its pharmacotherapy—increases the residual cardiovascular risk. Therefore, there is a clear need for proactive screening for cardiac diseases in psychiatric patients in general practitioners’ offices.

## Figures and Tables

**Table 2:** Risk of selected CVD in AN, their possible mechanisms and explanations.

Increased Risk in AN	Increased Risk Not Found in AN
Congestive heart failure due to decreased cardiac output and reduced LV mass, sometimes also observed in refeeding Conduction disorder due to electrolyte imbalance, changes in autonomic nervous system tone, and hormonal fluctuations Valvular disease with mitral valve prolapse is the most common, caused by valvulo-ventricular disproportion and decreased HRV Cardiomyopathy due to malnutrition and myocardial fibrosis, with cases of Takotsubo syndrome in AN patients being described Cardiac arrest is mostly caused by fluctuating potassium levels, hypoglycemia, and malignant arrhythmias	Stroke risk has been cited as only minimally higher than in the general population, with certain case studies presenting the occurrence of ischemic stroke only in extreme malnutrition in AN Atherosclerosis is not often observed in AN patients, probably due to the lack of risk factors in this group of individuals, with intima-medial thickness being similar to that of a healthy cohort Ischemic heart disease is associated with obesity and hypercholesterolemia, with the most likely cause being atherosclerosis, which is not a common occurrence in AN Inflammatory heart disease does not seem to manifest more frequently in AN patients despite some proinflammatory (mainly IL-6 and TNF-alpha) markers being elevated and causing structural changes in the myocardium

**Table 3:** Comparison between antidepressant groups and their impact on CVD risk and the cardiovascular system [104,105,110,111,112,113,114,115,116,117].

SSRIs	SNRIs	TCAs	Atypical Antidepressants
Generally considered the safest option in both cardiology and non-cardiology patients Certain evidence suggests higher stroke risk in SSRI users, although SSRIs still should be favored over TCAs in at-risk patients Sertraline most likely has either a positive effect on the cardiovascular system or no effect at all Citalopram and escitalopram carry a dose-dependent risk of QTc prolongation, so it is advised to monitor ECG in higher-dose treatment plans in patients above 65 y.o., cardiology patients, or those with impaired liver function Fluoxetine most probably has minimal positive effect on CVD risk and the heart, being more of a neutral choice and even potentially lowering MI risk, being a good choice in post-MI patients suffering from mild depression	Considered cardiologically neutral, with certain medications having minimal positive impact on cardiological outcomes Should be considered as a substitute for TCAs if TCAs are indicated Can be considered as a substitute for SSRIs if SSRIs are contraindicated or not effective Should be used with caution in HF patients Venlafaxine could be a potential sertraline replacement, though BP and HR monitoring is advised in higher-dose treatments Duloxetine appears to have minimal impact on RR, QRS, and QT intervals and does not pose a significant CVD risk in cardiology patients	Due to sodium and potassium channel blockage, they do possess dose-dependent cardiotoxic effects, including arrhythmias, QTc interval prolongation, and decreased contractility Adverse cardiac events were reported in people with no cardiological history Certain evidence shows that TCA use, as well as an abrupt TCA discontinuation, are both associated with stroke recurrence Higher doses were linked to a higher incidence of sudden cardiac death Nortriptyline use has been shown to be linked to a higher number of adverse cardiac events compared to paroxetine Use should be limited and preferably adequately consulted, especially in patients with cardiological history or post-stroke	Generally not recommended in patients with CVD risk factors or with cardiological history Trazodone has quite consistent evidence of having an arrhythmogenic effect; however, can be taken into consideration as a TCA replacement in certain scenarios, having an overall safer cardiological profile Mirtazapine does lead to weight gain and can potentially cause changes in HRV, although generally considered safe in short-term treatment in at-risk patients Bupropione does not seem to negatively affect the cardiovascular system, making it a potentially viable option for cardiology patients, especially while trying to quit smoking Other drugs, such as agomelatine or vortioxetine, have limited data on their CVD risk and use in at-risk patients but are not regarded as cardiotoxic as TCAs

**Table 4:** Overview of cardiovascular implications of the most often used medications in each category of psychotropics [104,111,116,122,126,127,128,129].

Antidepressants	Anxiolythics	Antipsychotics
Sertraline may lead to weight gain and have a slightly negative impact on lipidograms, although it is still the favored antidepressant in cardiology patients, even post-MI High doses of escitalopram and citalopram have been linked to QTc interval prolongation Citalopram is regarded as having the highest dose-dependent cardiotoxicity, possibly leading to sinus bradycardia and tachycardia, left and right bundle branch block, supraventricular tachycardia, and ventricular fibrillation Fluoxetine in high dosages poses a slight risk of QTc prolongation Venlafaxine can mildly increase BP and HR values Duloxetine might elevate BP mainly at the beginning of therapy, with BP values stabilizing later on Nortriptyline has a high risk of ECG changes, including QTc prolongation Trazodone may cause mild QTc prolongation and, when used in high doses, carries a risk of orthostatic hypotension Mirtazapine can potentially cause torsade de pointes (TdP) when combined with other TdP-inducing drugs and in at-risk groups	Benzodiazepines lower BP and HR Benzodiazepines are characterized by dose- and time-dependent cardiotoxicity—the bigger the dose and the time of prescription, the higher the risk of cardiovascular complications Low doses and short-term use of benzodiazepines exert a somewhat cardioprotective effect Z-hypnotics have similar CVD risk results as BDZs	Clozapine poses the highest risk of weight gain, type 2 diabetes, and dyslipidemia Olanzapine has a very high potential to cause weight gain, hyperglycemia, dyslipidemia, and type 2 diabetes Aripiprazole is associated with a higher risk of major adverse cardiovascular events in patients with type 2 diabetes and schizophrenia compared to risperidone Risperidone increases the risk of stroke in patients with dementia and a prior history of cerebrovascular events and in patients without known CVDs Ziprasidone carries a high risk of significant QTc prolongation Haloperidol in high doses may lead to QTc prolongation, is associated with ischemic heart disease in women, and, when administered intramuscularly, can cause orthostatic hypotension

## Data Availability

No new data were created or analyzed in this study.
